# Prospective validation of an artificial intelligence assessment in a cohort of applicants seeking financial compensation for asbestosis (PROSBEST)

**DOI:** 10.1186/s41747-025-00619-5

**Published:** 2025-08-15

**Authors:** Illaa Smesseim, Kevin B. W. Groot Lipman, Stefano Trebeschi, Martijn M. Stuiver, Renaud Tissier, Jacobus A. Burgers, Cornedine J. de Gooijer

**Affiliations:** 1 Department of Thoracic Oncology, Netherlands Cancer Institute, Amsterdam, The Netherlands; 2 GROW School for Oncology and Developmental Biology, Maastricht University Medical Center, Maastricht, The Netherlands; 3 Department of Radiology, The Netherlands Cancer Institute, Amsterdam, The Netherlands; 4 Division of Psychosocial Research and Epidemiology, Netherlands Cancer Institute, Amsterdam, The Netherlands; 5 Department of Epidemiology and Data Science, Amsterdam UMC, APH, University of Amsterdam, Amsterdam, The Netherlands

**Keywords:** Artificial intelligence, Asbestosis, Lung disease (interstitial), Pneumoconiosis, Pulmonologists

## Abstract

**Background:**

Asbestosis, a rare pneumoconiosis marked by diffuse pulmonary fibrosis, arises from prolonged asbestos exposure. Its diagnosis, guided by the Helsinki criteria, relies on exposure history, clinical findings, radiology, and lung function. However, interobserver variability complicates diagnoses and financial compensation. This study prospectively validated the sensitivity of an AI-driven assessment for asbestosis compensation in the Netherlands. Secondary objectives included evaluating specificity, accuracy, predictive values, area under the curve of the receiver operating characteristic (ROC-AUC), area under the precision-recall curve (PR-AUC), and interobserver variability.

**Materials and methods:**

Between September 2020 and July 2022, 92 adult compensation applicants were assessed using both AI models and pulmonologists’ reviews based on Dutch Health Council criteria. The AI model assigned an asbestosis probability score: negative (< 35), uncertain (35–66), or positive (≥ 66). Uncertain cases underwent additional reviews for a final determination.

**Results:**

The AI assessment demonstrated sensitivity of 0.86 (95% confidence interval: 0.77–0.95), specificity of 0.85 (0.76–0.97), accuracy of 0.87 (0.79–0.93), ROC-AUC of 0.92 (0.84–0.97), and PR-AUC of 0.95 (0.89–0.99). Despite strong metrics, the sensitivity target of 98% was unmet. Pulmonologist reviews showed moderate to substantial interobserver variability.

**Conclusion:**

The AI-driven approach demonstrated robust accuracy but insufficient sensitivity for validation. Addressing interobserver variability and incorporating objective fibrosis measurements could enhance future reliability in clinical and compensation settings.

**Relevance statement:**

The AI-driven assessment for financial compensation of asbestosis showed adequate accuracy but did not meet the required sensitivity for validation.

**Key Points:**

- We prospectively assessed the sensitivity of an AI-driven assessment procedure for financial compensation of asbestosis.
- The AI-driven asbestosis probability score underperformed across all metrics compared to internal testing.
- The AI-driven assessment procedure achieved a sensitivity of 0.86 (95% confidence interval: 0.77–0.95). It did not meet the predefined sensitivity target.

**Graphical Abstract:**

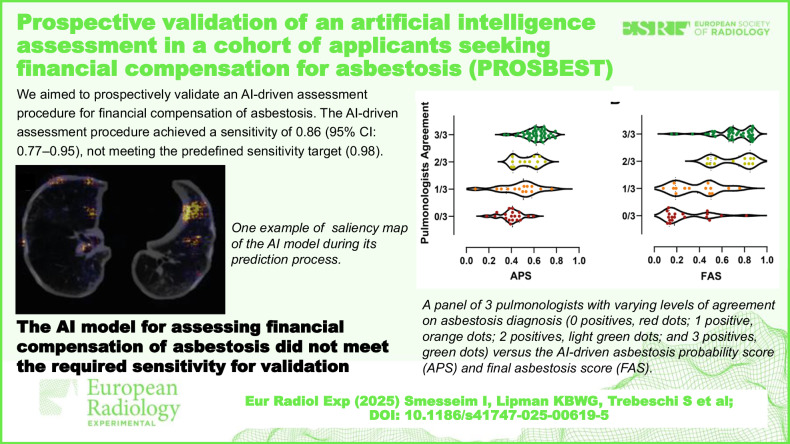

## Background

Asbestosis is a rare pneumoconiosis with diffuse pulmonary fibrosis emerging from extensive asbestos exposure [1]. In 2017, an incidence of 0.14 (uncertainty interval: 0.13–0.16) asbestosis cases per 100,000 inhabitants was reported in Western Europe, and asbestosis comprised 15.7% of all pneumoconiosis cases worldwide [2, 3]. The disease predominantly affects men, with a previous study reporting a prevalence of 78.1% [3]. Traditionally, the Helsinki criteria guide the diagnosis by integrating occupational asbestos exposure, latency, clinical signs, radiological findings, and lung function while excluding alternative causes [4, 5]. However, the invasive nature of definitive diagnostics such as lung biopsies hinders objectivity in the assessment [4], leading to variability in the interpretation of the disease. This underlines the need for innovation in diagnosis.

Previously, an automatic diagnostic method was developed combining an artificial intelligence (AI) classification for computed tomography (CT) with the diffusing capacity of the lung for carbon oxide (DLCO) [6].

An AI-driven asbestosis probability score was calculated to estimate the chance of a positive verdict to endorse financial compensation for asbestosis. This AI-supported assessment procedure showed significant discriminative power with an area under the curve of the receiver operating characteristic (ROC-AUC) of 0.95 (*p* < 0.0001). However, deployed AI models should demonstrate robust performance on prospective data rather than solely on data originating from the same timeframe as their training set, making prospective validation of AI models essential [7].

The primary objective of this study was to prospectively validate the sensitivity of the AI-driven assessment procedure in supporting financial asbestosis compensation cases in the Netherlands to assess whether this AI-driven assessment can be reliably applied in the real-world setting [6]. Secondary objectives were to assess the specificity, accuracy, positive predictive value (PPV), negative predictive value (NPV), ROC-AUC, and area under the precision-recall curve (PR-AUC) of the AI-driven assessment procedure.

## Materials and methods

To assess the sensitivity of the AI-driven assessment procedure in endorsing financial asbestosis compensation cases in the Netherlands, we prospectively included all adult patients who applied for asbestosis compensation to the Institute for Asbestos Victims (IAS) between September 2020 and July 2022. Patients who had already received financial compensation for mesothelioma were excluded, as patients in the Netherlands who have already received compensation for an asbestos-related disease are not eligible for additional compensation for another asbestos-related disease. Also, no proven asbestos exposure or applicants who had been non-Dutch residents for more than ten years were excluded. These are criteria established in the Dutch legislation regarding compensation for asbestos-related diseases. All eligible applicants underwent both the AI-driven index test and the standard review by three pulmonologists (reference test). All applications were evaluated by reviewers selected from a group of thirteen Dutch pulmonologists with more than five years of experience managing patients with asbestos-related diseases.

### AI-driven index test

The AI-driven index test consisted of an AI-supported assessment procedure that combined an AI classification for CT with the patient’s diffusing capacity of the lung for carbon monoxide (DLCO) and generated an AI-driven asbestosis probability score ranging from 0 (certainly not asbestosis) to 100 (certainly asbestosis). The AI-supported assessment procedure used in this study was previously developed in a research setting and has not been approved as a medical device for use in clinical practice [6]. For technical details, we refer to the main paper and supplemental materials of the original publication [6]. If the CT scan was incompatible or DLCO values were missing, an alternative formula was used (see Supplemental Material D). The scores were grouped into three categories:

- < 35 = Negative AI-driven index test.
- 35–66 = Undefined AI-driven index test.
- ≥ 66 = Positive AI-driven index test.

The AI-driven index test was considered as immediately positive if the AI-driven asbestosis probability score was *≥* 66.0 and negative if *<* 35.0. These cutoffs were based on a previous publication that showed the AI system achieved 100% accuracy for scores below 35 and above 60 on the test set [6]. A higher upper limit was chosen to allow for closer examination of the barely acceptable range (60–66). An uncertain AI-driven asbestosis probability score (35–66) triggered an assessment by two new independent reviewers to complete the index test. Their assessments were combined with the AI-driven asbestosis probability score to calculate the AI-driven final asbestosis score, as follows: (AI-driven asbestosis probability score + individual reviewer-1score + Individual reviewer-2 score)/3. For this final asbestosis score, a value of 0 was equal to “negative for asbestosis” and a value of 1 was equal to “positive for asbestosis”. The alternative formula, used in cases of incompatible CT scans or missing DLCO values (see Supplemental Material D), has not been previously validated. It was applied as it closely reflects real-world clinical practice.

Adjusting the automatic acceptance cutoff from 66 to original 60, we observed a sensitivity of 0.88 (95% confidence interval (CI): 0.79–0.96), and a specificity of 0.79 (0.68–0.94), with an accuracy of 0.85 (0.78–0.92), a ROC-AUC of 0.89 (0.80–0.96, *p* < 0.0001), a PR-AUC of 0.92 (0.84–0.98), a PPV of 0.88 (0.81–0.97), and an NPV of 0.79 (0.65–0.92). In total, 15 of 92 patients had an AI-driven asbestosis probability score of 60–66, and 3 of these 15 patients had a negative final asbestosis score.

In cases of an undefined AI-driven index test (*i.e*., with values from 35 to 66), two independent pulmonologists reviewed the cases. The AI-driven asbestosis probability score was then combined with the pulmonologists’ evaluations, and a final asbestosis score was calculated (see Supplemental Material D for formulas). A final asbestosis score ≥ 50 was considered positive, and a final asbestosis score < 50 was considered negative.

**Fig. 1:**
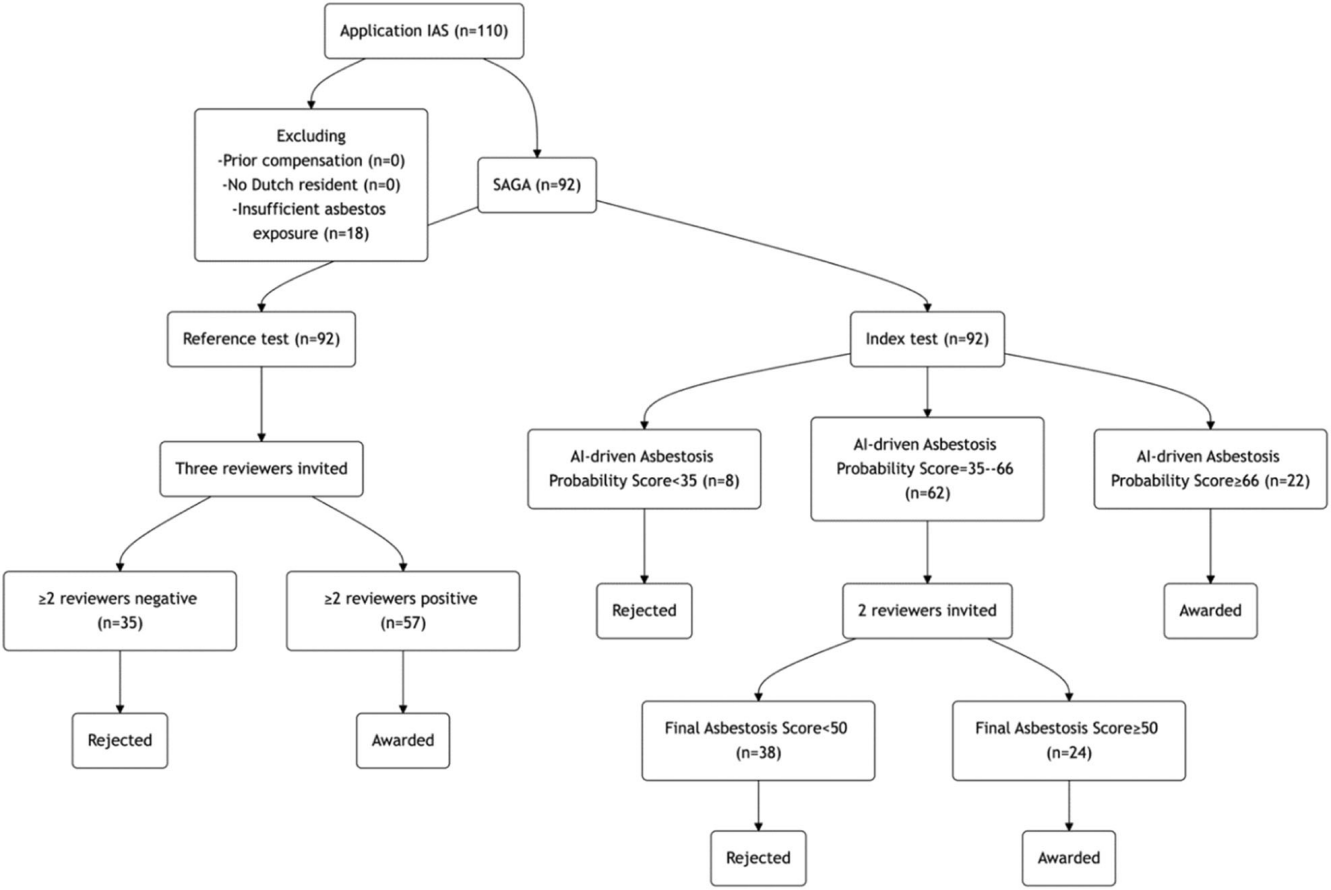
Study flowchart. IAS, Institute for Asbestos Victims; SAGA, Section of asbestos-related conditions

The reference test followed conventional evaluation methods, where three independent pulmonologists were pseudorandomly selected to assess each application. The reviewers were reassigned to different groups every three months based on a pre-established draw decision tree (see Supplemental Material G). Each case was evaluated according to the Dutch Health Council criteria for asbestosis [8]. A diagnosis of asbestosis requires at least two positive evaluations. All data were centrally processed using a national electronic case report form. If a patient was denied financial compensation and later submitted a reapplication with new medical information to support the asbestosis diagnosis, this reapplication was considered a new case. An overview of the study design is provided in Fig. 1.

All independent reviewers were blinded to whether they were performing an assessment for the reference test or the AI-driven index test. They were also blinded to the other pulmonologists’ verdicts and AI-driven asbestosis probability score or final asbestosis score. Randomization was performed by a randomization program in ALEA Clinical | FormsVision [9]. The reviewers were reallocated to different groups every three months. See Supplemental Material G for the decision tree. Three numbers were randomly drawn for the three reviewers of the reference test for every participant. The other numbers were assigned to the index test in case two additional reviewers were invited.

Applicants signed informed consent for using their data for scientific purposes, and the trial was registered at the Netherlands Trial Register as PROSBEST, Trial NL9064 [10]. The study protocol was exempted from ethical approval by our Institutional Review Board, as the Dutch Medical Research Involving Human Subjects Act [11] does not apply to this prospective observational study. This study is reported in accordance with the Standards for Reporting of Diagnostic Accuracy Studies guidelines [12, 13].

### Statistical analysis

A calculated sample size, determined through power analysis, ensured statistical significance for detecting the predefined 98% sensitivity level with a lower bound of 85% (see Supplemental Material A). Consequently, the number of positive applications following the reference test dictated the inclusion stop. We stated upfront that a false negative assessment would not have clinical consequences for the patient but would have financial implications, as compensation depends on it. Therefore, we aimed for a high NPV, which depends on sensitivity and the prevalence of the disease. This translated into a sensitivity target of 98%. Based on the anticipated sensitivity of 98% (95% CI: 85–100%) with a power of 0.99 in a one-sided test, the success criterion was met when 55 out of 59 patients were correctly classified as positive by the AI-driven index test (sensitivity = 93%, 95% CI: 85–100%). For reproducibility, our code for the power calculation is available online (https://github.com/nki-radiology/PROSBEST), open upon acceptance.

We evaluated the performance of the AI-driven index test by calculating sensitivity, specificity, accuracy, PPV, NPV, ROC-AUC, and PR-AUC. Bootstrapping to determine the CI was performed by in-place sampling with 10,000 iterations.

For the exploratory endpoint, *i.e*., the interobserver agreement, we considered that an AI tool can theoretically perform up to the interobserver agreement of the medical experts on the panel, since the labels in the training set of the AI are not a true reflection of the underlying disease, but a noninvasive surrogate, in or case the verdict of the panel. To study the impact of this phenomenon, the interobserver agreement of the panel was analyzed through the Randolph κ [14]. Unlike the Fleiss κ, which accounts for the distribution of verdicts in the dataset, the Randolf κ assigns the chance of diagnosing asbestosis at 0.5. We calculated the κ value for the entire population and per-subgroup, based on the AI-driven asbestosis probability score. Moreover, we calculated the κ value for all five medical experts for the applicants where the AI-driven asbestosis probability score was uncertain, and additional experts had to be consulted. The 95% CI was calculated through bootstrapping with replacement.

## Results

**Fig. 2:**
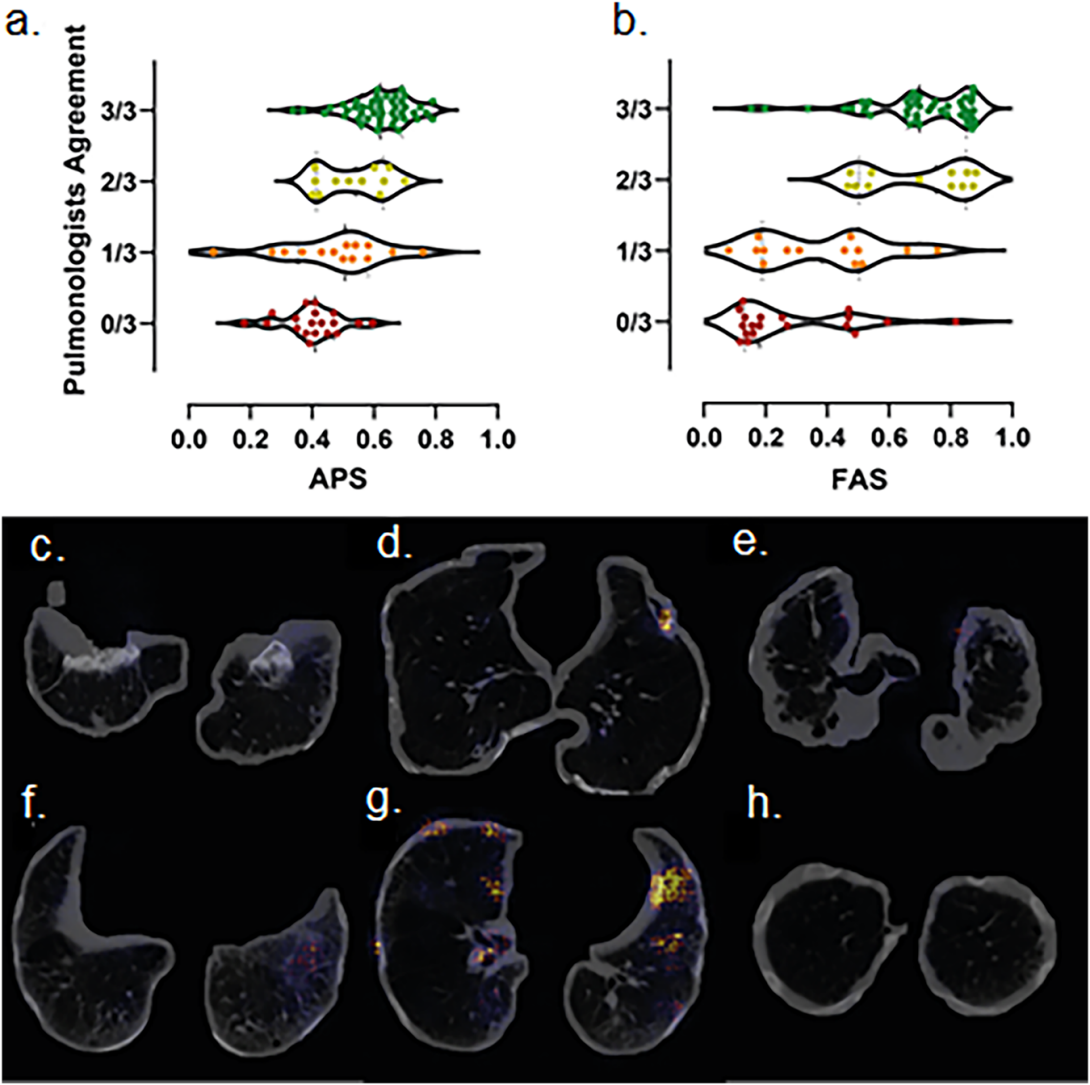
The concurrence among a panel of pulmonologists, with colors representing varying levels of agreement on asbestosis diagnosis: negative (red dots), one of three positive (orange), two of three positive (light green), and positive (green dots). **a**, **b** Illustrate violin plots for distinct prediction configurations, with the *y*-axis denoting pulmonologist consensus and the *x*-axis representing predicted asbestosis probabilities based on AI-driven asbestosis probability score (**a**) and final asbestosis score (**b**). **c**–**h** Axial unenhanced images of two subjects arranged in rows, featuring basal (**c**, **f**), midsection (**d**, **g**), and apical (**e**, **h**) slices. Colored overlays depict the saliency map, which approximates the areas of focus for the AI model during its prediction process

Between July 2020 and August 2022, 110 participants in the Netherlands were screened, with 92 of them meeting the inclusion criteria. Eighteen applicants were excluded due to insufficient asbestos exposure (Fig. 1). Detailed demographic and clinical variables such as age, gender, smoking status, pack years, and long functional loss according to the American Medical Association class are provided in Table 1. An adjusted AI-driven asbestosis probability score was applied for three applicants with an incompatible CT scan and five with missing DLCO values. According to the AI-driven index test, 8/92 applicants (8.7%) with an AI-driven asbestosis probability score < 35 were deemed negative, and 22/92 applicants (23.9%) received a positive assessment (AI-driven asbestosis probability score ≥ 66). Without additional reviewer’s input, the AI-driven asbestosis probability score yielded a sensitivity of 0.86 (95% CI: 0.78–0.94), specificity of 0.70 (0.57–0.87), accuracy of 0.80 (0.72–0.88), ROC-AUC of 0.87 (0.78–0.94), PR-AUC of 0.92 (0.85–0.97), PPV of 0.84 (0.75–0.93) and NPV: 0.74 (0.57–0.87). The agreement between the reference test pulmonologists and the AI-driven asbestosis probability score is shown in Fig. 2a.

**Table 1:** Study cohort: basic characteristics

Total population	92 (100)
Age at baseline in years (median, IQR)	75.5 (71.0–81.0)
Gender (number, %)	
Male	91 (98.9%)
Female	1 (1.1%)
Smoking status (number, %)	
Non-smoker	9 (9.8%)
Former smoker	51 (55.4%)
Current smoker	3 (3.3%)
Missing	29 (31.5%)
Pack year (median, IQR)	28.0 (15.0–40.0)
Lung function loss, American Medical Association class	
0	8 (8.7%)
1	9 (9.8%)
2	18 (19.6%)
3	17 (18.5%)
4	38 (41.3%)
Missing	2 (2.2%)
Number of positive verdicts in the reference test	
0/3	19 (20.7%)
1/3	16 (17.4%)
2/3	12 (13.0%)
3/3	45 (48.9%)

Data are given as numbers (%) unless otherwise specified

*IQR* Interquartile range

The key difference between our study and the previous study [6] is that the AI tool was developed using a retrospectively collected cohort of Dutch patients who had applied for asbestosis compensation at the IAS, while the patients in the current study were included prospectively. However, the inclusion and exclusion criteria were the same in both studies.

Of 92 applicants with an AI-driven asbestosis probability score between 35 and 66, 62 (67.4%) were also reviewed by two additional reviewers. After their review, the AI-driven final asbestosis score resulted in 28/92 (30.4%) approved cases and 34/92 (37.0%) denied cases. This process achieved the following performance metrics: 0.86 (95% CI: 0.77–0.95) sensitivity, 0.85 (0.76–0.97) specificity, 0.87 (0.79–0.93) accuracy, 0.92 (0.84–0.97) ROC-AUC, 0.95 (0.89–0.99) PR-AUC, 0.91 (0.85–0.98) PPV and 0.78 (0.64–0.91) NPV. The agreement between the pulmonologists in the reference test and the AI-driven final asbestosis score is visualized in Fig. 2b.

The correlation between the AI-driven asbestosis probability score and the AI-driven final asbestosis score was τ = 0.49. After correcting for the correlation in assessments, the increase in ROC-AUC from adding reviewers was statistically significant (Equation 2, *p* = 0.021), see Supplemental Material B. Details of the 8/92 applicants with a negative AI-driven index test but a positive reference test are summarized in (Supplemental Material J). Interestingly in 2 cases, the reference panel was unanimously positive, the AI uncertain, and the two additional reviewers were unanimously negative (examples A and G).

Regarding the interobserver agreement, one case was excluded from analysis due to a missing reviewer’s verdict. We observed a Randolph kappa of the reference test (panel three pulmonologists) of 0.59 (CI: 0.46–0.71, *p* < 0.001, *n* = 91). When the cohorts were split on asbestosis probability score, we found a kappa of 0.50 (0.00–0.83, *p* < 0.001, *n* = 8) for the asbestosis probability score *<* 35. The uncertain group (35 *<* asbestosis probability score < 66) yielded a kappa of 0.52 (0.37–0.67, *p* < 0.001, *n* = 61). Including all *n* = 5 reviewers for this cohort, the kappa decreased to 0.42 (0.30–0.55, *p* < 0.001, *n* = 61). In the group that was immediately accepted (asbestosis probability score ≥ 66) in the index test, the kappa among pulmonologists in the reference test was 0.84 (0.61–0.92, *p* < 0.001, *n* = 17).

## Discussion

Our study demonstrated that, although the AI-supported assessment procedure for endorsing financial asbestosis compensation cases in the Netherlands achieved a sensitivity of 0.86 (95% CI: 0.77–0.95), it did not meet the predefined sensitivity target. The strength of this study lies in its prospective, double-arm design, which allowed a direct comparison between evaluations made by the conventional panel of three independent pulmonologists and the AI-driven index test.

Notably, the AI-driven asbestosis probability score underperformed across all metrics compared to internal testing. The significant level of interobserver variability between reviewers may have limited the performance of the AI arm. We observed three instances where pulmonologists assessing the AI-driven index test completely disagreed with those evaluating the reference test, which highlights the interobserver variability in this study and the clinical heterogeneity of asbestosis [15, 16].

Assigning pulmonologists to the reference test or AI-driven index test was random, which suggests that three pulmonologists per panel may be insufficient to overcome variability for cases deemed uncertain by the AI. This presents a trade-off between more automatic assessments at the cost of potentially granting unnecessary financial compensation. In follow-up studies, quantitative measures of interobserver variability should be included in the power analysis to allow for more flexible requirements, as no assessment tool (or expert) can meet strict criteria when the reference test itself contains substantial interobserver variability [17, 18].

Upon analyzing the kappa scores for variability, we observed moderate to substantial agreement within the reference panel. For the cohort that should be directly awarded compensation based on the AI-driven Asbestosis Probability, the agreement in the reference panel was moderate. When the AI-driven Asbestosis Probability was uncertain, the agreement was moderate for the reference panel and fair to moderate when including all five reviewers. Comparing kappa values between these groups is not possible due to the different numbers of reviewers. The agreement among five reviewers is likely a more accurate representation of the interobserver variability within the entire panel.

Even though the difference in performance was not statistically significant compared to the original study, the reduction in AUC is quite substantial. While not significant, it should be noted that the study was not powered to detect a reduction in performance. When the AI-driven asbestosis probability score directly determines compensation, moderate agreement is observed within the reference panel. Considering these observations, it may be useful to refine the guidelines for uncertain cases. Additionally, an automatic AI-supported assessment could be used to detect these uncertain cases.

A strength of our study is that the study population aligns with real-life data in the Netherlands. We included all patients who were registered for asbestosis compensation. During selection, we intentionally applied the legal inclusion and exclusion criteria set by the government for granting compensation for asbestosis-related diseases. Because the inclusion of our patients directly corresponds to how patients are registered for asbestosis compensation in real life, we have minimized the risk of selection bias.

Our study has some limitations. This study has been validated on the Dutch population. The question is whether the AI tool can also be used in populations of other countries due to differences in the epidemiology of asbestosis. One example is the prevalence of asbestosis among men, which was higher in our population than the previously reported prevalence in Western Europe. Another limitation was that since we were only interested in whether the sensitivity was not below a certain value, we opted for a one-sided test. For future research, we would recommend to choose for a two-sided test requiring a larger sample size. Also, the fact that the research team behind the development of the original AI tool was also partly involved in this prospective study may have led to bias. For future validation research, we recommend including researchers who were not involved in the development of the AI tool, with the aim of reducing the risk of biases such as confirmation bias. As asbestosis is a rare disease and expert reviewers are limited, we aim to conduct external validation in a different country for future research. In this setting, a new team of expert reviewers who were not involved in the development of the AI tool can assess the cases. External validation will also help determine whether our findings are applicable to other legal, clinical, or epidemiological contexts. Another limitation was that, for this study, the ground truth was determined by the evaluation of three reviewers. Although a clear ground truth is essential in AI development, in clinical practice, a definitive diagnosis is not always possible, particularly in the diagnostic process of interstitial lung diseases.

We consider lung biopsies to be too invasive and high-risk to serve as a tool for establishing a more robust ground truth. Hutchinson et al previously reported an in-hospital mortality rate of 16.0% for non-elective surgical lung biopsies performed in patients with interstitial lung disease [19]. To achieve a more reliable ground truth, we suggest increasing the number of reviewers, for example, to five. Currently, the model is trained on the (noisy) noninvasive diagnoses of the panel, which limits the model’s additional value [20, 21]. In the current setting, the AI program is not a suitable aid in the diagnostic workup of asbestosis. An option to improve its performance is to use an objective quantification of the fibrosis through segmentation to remove interobserver variability on the ‘5% diffuse fibrosis rule’ [16, 18]. AI has previously shown great potential in medical imaging in settings that rely on multiple readers [22, 23]. For future research, an alternative design could be investigated to evaluate diagnostic accuracy and workflow efficiency, where AI replaces one of the three human readers instead of functioning as a fully autonomous system. This approach would align better with the current interpretation of the EU AI Act and, at the same time, could help lower procedure costs while maintaining diagnostic sensitivity. Our study shows that developing an AI tool to help in the diagnostic process of asbestosis is challenging, and many hurdles must still be overcome before this AI tool can be implemented in clinical practice. In the future, we aim to improve the AI-driven decision-making process for applications for government support for asbestosis.

In conclusion, this study prospectively evaluated the sensitivity of an automatic assessment for asbestosis in a cohort of applicants seeking financial compensation for asbestosis through AI. The AI-driven index test did not meet the predefined criteria for successful validation. Retrospectively, the requirements were set unrealistically high for a disease with a wide range of clinical features. Further evaluation of the automatic assessment in the same or a broader clinical setting is warranted only if efforts are made to reduce interobserver variability, potentially through the implementation of stricter diagnostic criteria.

## Supplementary information

**Additional file 1: Supplement Table 1.** The reviewers were re-allocated to different groups every three months. Draw decision tree: three (form) numbers were randomly drawn for the three reviewers of the reference test for every participant. The other (form) numbers were assigned to the index test in case two additional reviewers were invited. **Supplement Table 2.** Examples of participants that were negative in the AI-driven index test and positive in the reference test. DLCO: diffusing capacity of the lungs for carbon monoxide in %. I-R: index test reviewer. R-R: reference test reviewer.

## Data Availability

The participants of this study did not give written consent for their data to be shared publicly, so the research supporting data is not available.

## Abbrevations

AI: Artificial intelligence
CI: Confidence interval
CT: Computed tomography
DLCO: Diffusing capacity of the lung for carbon oxide
IAS: Institute for Asbestos Victims
NPV: Negative predictive value
PPV: Positive predictive value
PR-AUC: Area under precision-recall curve
ROC-AUC: Area under the curve of the receiver operating characteristic
