# Supplementary material for: Prospective validation of an artificial intelligence assessment in a cohort of applicants seeking financial compensation for asbestosis (PROSBEST)
Source: Eur Radiol Exp. 2025 Aug 15;9:76. doi: 10.1186/s41747-025-00619-5 (PMC12356797; doi:10.1186/s41747-025-00619-5)
Supplement: Supplementary file 1 — Additional file 1: Supplement Table 1. The reviewers were re-allocated to different groups every three months. Draw decision tree: three (form) numbers were randomly drawn for the three reviewers of the reference test for every participant. The other (form) numbers were assigned to the index test in case two additional reviewers were invited. Supplement Table 2. Examples of participants that were negative in the AI-driven index test and positive in the reference test. DLCO: diffusing capacity of the lungs for carbon monoxide in %. I-R: index test reviewer. R-R: reference test reviewer. [file 41747_2025_619_MOESM1_ESM.pdf]

Prospective validation of an artificial intelligence assessment in a cohort of applicants seeking financial compensation for asbestosis (PROSBEST)

ELECTRONIC SUPPLEMENTARY MATERIAL

A. Sample size calculation

We computed the sample size based on a targeted sensitivity of 98%. Since we were only interested in whether the sensitivity was not below a certain value, we chose to test one-sided. Additionally, since the prevalence of asbestosis fluctuated per year in the applying population, and only asbestosis patients (classified as having asbestosis by the reference standard) contributed to the sensitivity, we opted for a power calculation based on the positive applications only. We chose to accept a large error because: (1) the reference test was subject to bias and (2) the false-negative cases would be evaluated before implementing the AI-driven assessment.

B. Z-value calculations

To test for significant differences between the original study's performance and the prospective trial, we calculated the z-value via equation 1. For comparison between the AI-driven Asbestosis Probability Score and Final Asbestosis Score of the index test, we adjusted for correlation in predictions via equation 2 [16].

$$z = AUC_1 - \frac{AUC_1 + AUC_2}{se_1^2 + se_2^2}$$

Equation 1: Where *AUC* is the ROC-AUC, *se* is the standard error of the ROC-AUC.

$$z = AUC_1 - \frac{AUC_1 + AUC_2}{sqrt(se_1^2 + se_2^2 - 2 * r * se_1 * se_2)}$$

Equation 2: Where *AUC* is the ROC-AUC, *se* is the standard error of the ROC-AUC, and *r* is the correlation via Kendall's tau (τ).

C. AI program cutoffs

As secondary analysis, we calculated the sensitivity using the original cutoffs based on the earlier publication that demonstrated the AI system achieved 100% accuracy for AI-driven Asbestosis Probability Score <35 and >60 on the respective test set. To ensure that the AI is of value (i.e. the performance of the index test is not driven by additional reviewers), we evaluated the performance of the AI-driven Asbestosis Probability Score following the same performance criteria utilized for the Final Asbestosis Score.

D. Formulas for the index test

1. AI-driven Asbestosis Probability Score in percentage: range 0-100

    a) AI-driven Asbestosis Probability Score (if both lung function test and eligible CT are available)

$$\text{AI} - \text{driven Asbestosis Probability Score} = \frac{(100 * AI\ output\ on\ CT) + (100 - DLCO)}{2}$$

    b) AI-driven Asbestosis Probability Score (if only a lung function test is available) = $100 - DLCO$

    c) AI-driven Asbestosis Probability Score (if only an HRCT is available) = $100 * AI\ output\ on\ CT$

2. Individual Reviewer Score in percentage: range 0-100 (no: 0, yes: 100)

3. Final Asbestosis Score in percentage: range 0-100

4. $Final\ Asbestosis\ Acore =$

$$\frac{\text{AI−driven Asbestosis Probability Score } + Individual\ Reviewer\ Score\ 1 + Individual\ Reviewer\ Score\ 2}{3}$$

E. Informed consent

The trial was registered at the Netherlands Trial Register as PROSBEST, Trial NL9064 [5]. The study protocol was exempted from ethical approval by our institute's medical ethics committee, as the Medical Research Involving Human Subjects Act does not apply to this prospective observational study. Applicants signed informed consent for using their data for scientific purposes.

F. Availability of data and materials

Information was collected in an electronic Case Report Form (eCRF).

G. Randomisation

	Group A	Group B	Group C	Group D
Form 1	Reviewer 1	Reviewer 6	Reviewer 11	Reviewer 16
Form 2	Reviewer 2	Reviewer 7	Reviewer 12	Reviewer 17
Form 3	Reviewer 3	Reviewer 8	Reviewer 13	Reviewer 18
Form 4	Reviewer 4	Reviewer 9	Reviewer 14	Reviewer 19
Form 5	Reviewer 5	Reviewer 10	Reviewer 15	Reviewer 20

Supplement Table 1: The reviewers were re-allocated to different groups every three months. Draw decision tree: three (form) numbers were randomly drawn for the three reviewers of the reference test for every participant. The other (form) numbers were assigned to the index test in case two additional reviewers were invited.

H. Exact score calculation

If a CT scan was incompatible with the requirements of the AI model (i.e. slice increment ≥ 5mm), then only the DLCO was processed for that applicant. If the DLCO was missing, then only the AI output score on CT would determine the AI-driven Asbestosis Probability Score. In absence of a CT chest and lung function test, the application was automatically rejected, in line with the reference test. Combining the AI output score on the CT scan with the DLCO of the applicant resulted in the AI-driven Asbestosis Probability Score (APS) [4].

I. Statistical analysis

The AI-supported procedure would be successful for the asbestosis application process in The Netherlands if the entire one-sided exact 95% confidence interval around the sensitivity observed in our study lies above the boundary of 85%.

J. Details of applicant with a negative AI-driven Final Asbestosis Score (index test) but a positive reference test

#	DLCO	AI-driven index test	I-R1	I-R2	Final Asbestosis Score	R-R1	R-R2	R-R3
A	56	0.6	0	0	0.2	1	1	1
B	43	0.48	0	1	0.49	0	1	1
C	68	0.34	-	-	0.34	1	1	1
D	52	0.4	0	1	0.47	1	0	1
E	61	0.44	0	1	0.48	1	1	1
F	56	0.48	1	0	0.49	1	1	1
G	51	0.46	0	0	0.15	1	1	1
H	72	0.37	0	1	0.46	1	1	1

Supplement Table 2: Examples of participants that were negative in the AI-driven index test and positive in the reference test. DLCO: diffusing capacity of the lungs for carbon monoxide in %. I-R: index test reviewer. R-R: reference test reviewer.
